# Impairment of neutrophil functions and homeostasis in COVID-19 patients: association with disease severity

**DOI:** 10.1186/s13054-022-04002-3

**Published:** 2022-05-30

**Authors:** Chloé Loyer, Arnaud Lapostolle, Tomas Urbina, Alexandre Elabbadi, Jean-Rémi Lavillegrand, Thomas Chaigneau, Coraly Simoes, Julien Dessajan, Cyrielle Desnos, Mélanie Morin-Brureau, Yannick Chantran, Pierre Aucouturier, Bertrand Guidet, Guillaume Voiriot, Hafid Ait-Oufella, Carole Elbim

**Affiliations:** 1 INSERM, UMRS 938, Hôpital St-Antoine, Centre de Recherche Saint-Antoine, Sorbonne Université, 75012 Paris, France; 2 Sorbonne Université, Paris, France; 3 Service de Médecine Intensive-Réanimation, Hôpital Saint-Antoine, Assistance Publique-Hôpitaux de Paris, Paris, France; 4 Service de Médecine Intensive-Réanimation, Hôpital Tenon, Assistance Publique-Hôpitaux de Paris, Paris, France; 5 INSERM U970, Cardiovascular Research Center, Université de Paris, Paris, France; 6 Département d’Immunologie Biologique, Hôpital Saint-Antoine, Assistance Publique-Hôpitaux de Paris, Paris, France

**Keywords:** COVID-19, Neutrophil, Oxidative burst, Angiogenic neutrophils, Vascular inflammation

## Abstract

**Background:**

A dysregulated immune response is emerging as a key feature of critical illness in COVID-19. Neutrophils are key components of early innate immunity that, if not tightly regulated, contribute to uncontrolled systemic inflammation. We sought to decipher the role of neutrophil phenotypes, functions, and homeostasis in COVID-19 disease severity and outcome.

**Methods:**

By using flow cytometry, this longitudinal study compares peripheral whole-blood neutrophils from 90 COVID-19 ICU patients with those of 22 SARS-CoV-2-negative patients hospitalized for severe community-acquired pneumonia (CAP) and 38 healthy controls. We also assessed correlations between these phenotypic and functional indicators and markers of endothelial damage as well as disease severity.

**Results:**

At ICU admission, the circulating neutrophils of the COVID-19 patients showed continuous basal hyperactivation not seen in CAP patients, associated with higher circulating levels of soluble E- and P-selectin, which reflect platelet and endothelial activation. Furthermore, COVID-19 patients had expanded aged-angiogenic and reverse transmigrated neutrophil subsets—both involved in endothelial dysfunction and vascular inflammation. Simultaneously, COVID-19 patients had significantly lower levels of neutrophil oxidative burst in response to bacterial formyl peptide. Moreover patients dying of COVID-19 had significantly higher expansion of aged-angiogenic neutrophil subset and greater impairment of oxidative burst response than survivors.

**Conclusions:**

These data suggest that neutrophil exhaustion may be involved in the pathogenesis of severe COVID-19 and identify angiogenic neutrophils as a potentially harmful subset involved in fatal outcome.

**Graphic Abstract:**

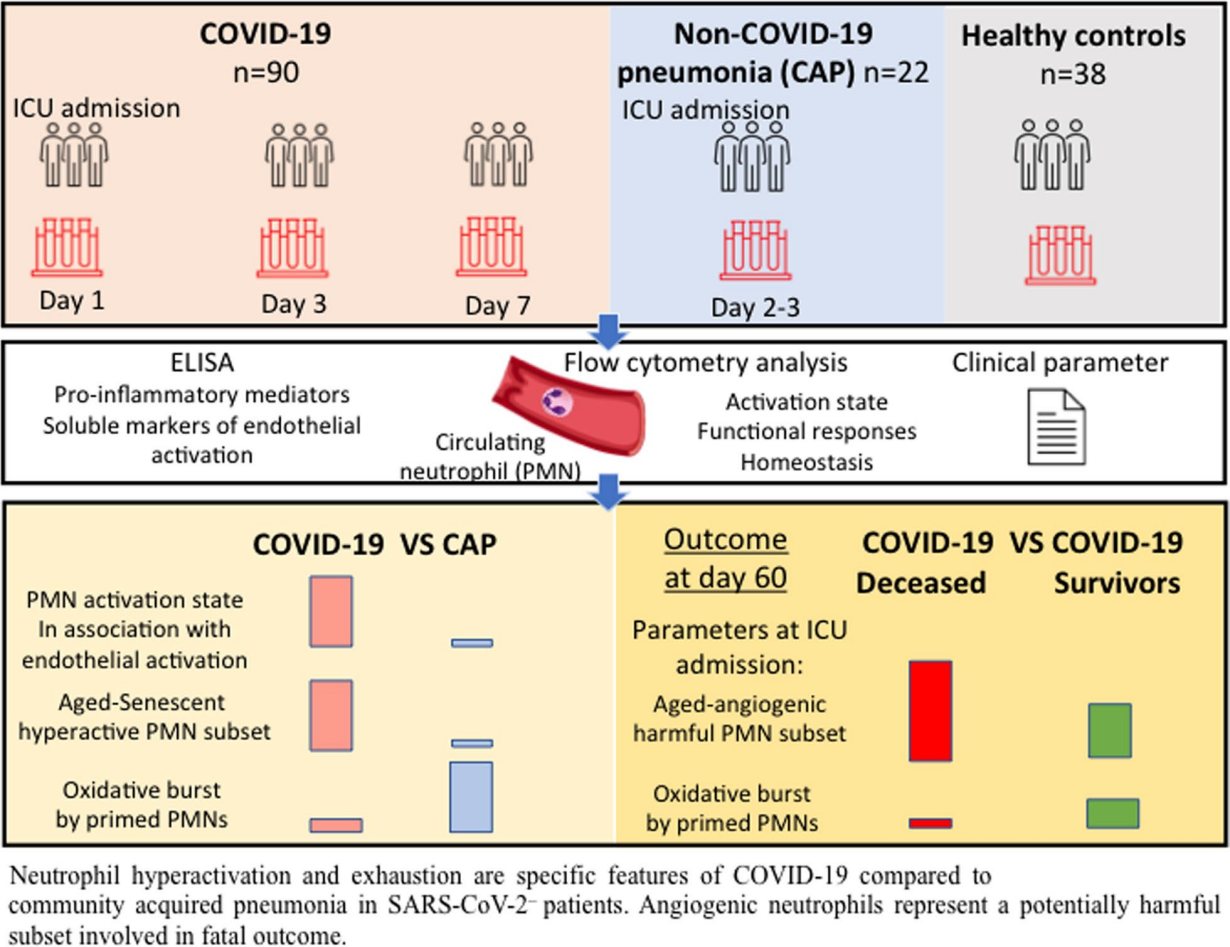

**Supplementary Information:**

The online version contains supplementary material available at 10.1186/s13054-022-04002-3.

## Background

The rising pandemic of coronavirus disease 2019 (COVID-19) caused by severe acute respiratory syndrome coronavirus 2 (SARS-CoV-2) has led to worldwide economic harm and deaths. The spectrum of clinical manifestations in SARS-CoV-2-infected patients (SARS-CoV-2^+^) ranges from asymptomatic to severe acute respiratory distress syndrome (ARDS) and multiple organ involvement [1]. Emerging data and clinical reports increasingly suggest that severe COVID-19 reflects a confluence of micro-vascular damage related to endothelial dysfunction and/or impaired angiogenesis, and dysregulated inflammation [2].

Neutrophils, the most abundant leukocytes in the blood, are known for providing immediate frontline protection against rapidly dividing bacteria and fungi. A growing body of evidence implicates neutrophils, via their generation of reactive oxygen species (ROS), neutrophil extracellular traps (NETs), and ability to act as antigen-presenting cells, in the host response to viral infections [3, 4]. However, the inappropriate activation of neutrophils can lead to oxidative stress and uncontrolled systemic inflammation that damage the capillary endothelium and disrupt the thrombo-protective state of endothelial cells [5]. Recent data have shown that polymorphonuclear neutrophils are functional, versatile, and phenotypically diverse [6]. Specific subpopulations of neutrophils that have great tissue-destructive potential are implicated in endothelial dysfunction, angiogenesis, and vascular inflammation: (i) senescent neutrophils become overactive, produce strong ROS responses, and express high levels of CXCR4 [7]; (ii) neutrophils have been observed to perform reverse transendothelial migration (rTEM), re-entering the circulation and then potentially spreading throughout the body via the bloodstream, transmigrating into other organs and contributing to more injuries to more organs and to systemic inflammation [8]; (iii) pro-angiogenic neutrophils [9] are reported to migrate to hypoxic tissue and participate in neovascularization [10].

Studies conducted during the first COVID-19 wave reported the robust generation of NETs in patients with severe COVID-19 [11, 12], the presence in the circulation of immature and suppressive myeloid cells, including neutrophils, as well as neutrophilic infiltrates in the lung [13–19]. In addition, although substantial evidence points toward a vascular disease process as a contributor to COVID-19 pathogenesis, no data about senescent, rTEM, or angiogenic neutrophils in COVID-19 patients have been reported. Finally, as the success of dexamethasone treatment in oxygen-dependent COVID-19 patients might be explained by its anti-inflammatory as well as its clear vasoconstrictive effects, there is a real need to re-evaluate the neutrophil compartment taking this treatment into account.

We characterized in detail the phenotypes and longitudinal functions of fresh whole-blood circulating neutrophils in a large cohort of severely ill COVID-19 patients (*n* = 90) in the ICU receiving steroid therapy, comparing them with those of 22 SARS-CoV-2^–^ patients hospitalized for severe community-acquired pneumonia (CAP) and 38 healthy controls similar for sex and age. We also assessed correlations between these phenotypic and functional indicators and markers of endothelial damage as well as disease severity.

## Materials and methods

### Study design

This study enrolled 90 COVID-19 patients admitted to the intensive care units (ICUs) of Saint-Antoine and Tenon Hospitals (Paris, France) with moderate-to-severe ARDS according to the Berlin definition [20] and SARS-CoV-2 infection confirmed by reverse transcription polymerase chain reaction (RT-PCR) tests of nasopharyngeal swab samples. At admission, we prospectively collected the following data for each: demographic information, including age, sex, body mass index (BMI), comorbidities according to the Charlson index, dates of first symptoms, hospital and ICU admissions, and vital signs. The SOFA score was calculated at admission and every 3 days until discharge or death. The following data regarding medical management in the ICU were collected daily: mechanical ventilation settings after intubation (mode, PEEP, FiO_2_, respiratory rate, tidal volume, and plateau pressure), duration of mechanical ventilation, use of advanced therapies for acute respiratory failure (neuromuscular blocking agents, inhaled pulmonary vasodilators, prone positioning, and extracorporeal membrane oxygenation), antiviral therapies and immunomodulatory agents (i.e., interleukin-6-receptor antagonists and corticosteroids) with time from symptom onset to initiation, and any acute kidney injury, acute cardiac injury, pulmonary embolism or deep venous thrombosis.

A second cohort included 22 patients admitted to Saint-Antoine and Tenon Hospitals ICU with non-SARS-CoV-2 community-acquired pneumonia (CAP). All episodes of pneumonia were classified as severe and required invasive mechanical ventilation. Pneumonia severity was assessed through the SOFA score and the Pneumonia Severity Index. A third cohort consisted of 38 age-matched healthy controls (HCs), with blood biochemical and hematological values within normal range.

Whole blood was sampled, kept on ice, and transported immediately to the laboratory for neutrophil analysis as previously described [21]. COVID-19 patients provided samples at their inclusion on ICU admission (Day 1). Analysis at day 1 was performed a median of 10 days after the onset of symptoms. When possible, follow-up samples were obtained at 3 days and at 7 days after the baseline sample (Day 1) for COVID-19 patients. CAP patients gave a single blood sample at their inclusion on ICU admission, and HCs also donated blood only once.

### Determination of neutrophil subsets

The neutrophil subsets were assessed by using 10-color flow cytometry (Gallios Flow Cytometer; Beckman Coulter, Fullerton, Calif). The detailed staining procedure is described in the Additional file 1: Methods.

### Determination of adhesion molecule expression on resting and stimulated neutrophils

Heparin whole-blood samples (500 μL) were either kept on ice or incubated with PBS or 10^−6^ M bacterial peptide formyl-methionyl-leucyl-phenyl-alanine (fMLP) (Sigma Chemical Co., St Louis, MO) for 5 min. Samples were stained with PE-anti-human CD11b (clone 2LPM19c, Dakopatts, Glostrup, Denmark) and APC-anti-human CD62L (clone DREG-56, BD Biosciences) as previously reported [22]. Samples were then analyzed by means of flow cytometry, as described in the Additional file 1: Methods.

### Measurement of neutrophil oxidative burst

Superoxide anion (O_2_^−^) production by neutrophils was measured with a flow cytometry-based assay derived from the hydroethidine (HE) oxidation technique, as previously described [22]. Heparinized whole-blood samples (500 μL) were loaded for 15 min with 1500 ng/mL HE (Sigma Chemical Co., St Louis, MO) at 37 °C and then incubated for 45 min at 37 °C with PBS, TNF-α (5 ng/mL, R&D Systems, Minneapolis, MN), lipopolysaccharide (LPS) from *E. coli* serotype R515 (TLR4 agonist, 10 ng/mL, Alexis Biochemicals, San Diego, CA) or ssRNA with 6 UUGU repeats/LyoVec™ (TLR8 Agonist, 50 μg/mL, InvivoGen, Toulouse, France). Samples were then treated with PBS or 10^−6^ M fMLP (Sigma Chemical Co., St Louis, MO) for 5 min. Samples were then analyzed by means of flow cytometry, as described in the Additional file 1: Methods.

### Determination of neutrophil-platelets aggregates

For the analysis of neutrophil-platelet aggregates, whole-blood samples collected on anticoagulant citrate-dextrose solution (ACD) were incubated for 45 min with PE-anti-human CD16b (clone CLB-gran11.5, BD Biosciences), APC-anti-human CD15 (clone HI98, BD Biosciences) and FITC-anti-human CD41b (clone HIP2^23^]. Staining with anti-CD15 monoclonal antibody allowed us to identify PMNs in whole blood on the CD15/side scatter dot plot and to gate out other cells, erythrocytes, and debris. Fluorescence analysis was performed on this gate. Neutrophil-platelet aggregates were identified as the percentages of CD16b^+^CD41^+^ cells in the gated neutrophil population. Fluorescence of isotypic controls served as negative controls.

### Measurement of soluble pro- and anti-inflammatory mediators

Whole-blood samples were centrifuged for 15 min at 1000*g* within 30 min of collection. Soluble cytokines (IL-6, IL-10 and TGFβ), junctional adhesion molecule (JAM-)C, LTB4, neutrophil elastase (NE), E-selectin, P-selectin, VEGF, and VEGF-R1 were detected from serum by ELISA according to the appropriate dilution and following recommendations of the manufacturer (Human IL-6 Quantikine ELISA kit, R&D Systems, Catalog number: D6050; Human IL-10 Quantikine ELISA kit, R&D Systems, Catalog number: D1000B; Human TGFβ1 ELISA Kit, Abcam, Catalog Number: AB100647; Human JAM-C ELISA kit, Sigma Aldrich, Catalog number: RAB1067; Human LTB4 Parameter Assay kit kit, R&D Systems, Catalog number: KEG006B; Human Neutrophil Elastase SimpleStep ELISA Kit, Abcam, Catalog Number: ab270204; Human E-Selectin/CD62E Immunoassay, R&D Systems, Catalog Number: DSLE00; Human P-Selectin/CD62P Immunoassay, R&D Systems, Catalog Number: DPSE00; Human VEGF Quantikine ELISA Kit, R&D Systems, Catalog Number: DVE00; Human VEGFR1/Flt-1 Quantikine ELISA Kit, R&D Systems, Catalog Number: DVR100C). Cryostored samples frozen at − 80 °C and diluted according to the manufacturer’s instructions were assayed (R&D Systems).

### Statistics

The freely available software Rstudio 1.0.143 (http://www.rstudio.com/) was used for statistical analysis. All tests were two-tailed, with a significance level of *α* = 0.05. When a parametric test was used, normality of distribution was tested with the Shapiro–Wilk test. Differences between groups were assessed with the chi-square test or ANOVA, followed by the Tukey post-hoc test, as appropriate. ANOVA, adjusted for age, was used to compare neutrophil markers between the COVID-19 groups and controls (with age as a covariate). Bonferroni correction was used for multiple comparisons. Linear partial correlation analysis, with adjustment for age, identified correlations.

## Results

### Demographics and baseline characteristics of COVID-19 and CAP patients

Clinical and biological characteristics of the 90 COVID-19 and the 22 CAP patients are shown in Table 1 and Additional file 1: Table S1. All patients received oxygen therapy and antibiotics during hospitalization. 50% required invasive mechanical ventilation, and 4.3% extracorporeal membrane oxygenation. Most hospitalized patients had at least one comorbidity, regardless of SARS-CoV-2 status. A lower proportion of SARS-CoV-2^–^ subjects had comorbidities associated with a risk of severe COVID-19 compared with SARS-CoV-2^+^ patients. Among SARS-CoV-2^−^ subjects, at least one bacterium was identified in 14 (64%) patients and at least one virus in 8 patients (36%). S*treptococcus pneumoniae* was the was the most commonly identified bacterium found in 8 patients (36%). Taken together, *Legionella pneumophilia*, *Haemophilus influenzae*, *Pseudomonas aeruginosa* and *Enterobacteriaceae species* were identified in 8 (36%) patients of the bacterial group. Influenza viruses were the most commonly identified viruses, found in 6 (21.8%) patients.

**Table 1:** Characteristics of COVID-19 patients and SARS-CoV-2^–^ patients hospitalized for severe community-acquired pneumonia (CAP) participating in the study

Characteristics	COVID-19	CAP	*P* value
	*N* = 90	*N* =	
Men (*N*, %)	57 (63%)	14 (64%)	NS
Age (years, SD)	63 ± 11	69 ± 17	0.0171
SOFA score Day 1	3.5 [0–12]	6 [1–13]	0.0065
*Body mass index*			
< 30	47 (52%)	16 (73%)	NS
> 30	40 (44%)	5 (25%)	NS
*Comorbidity (N, %)*			
Arterial hypertension	60 (67%)	10 (45%)	NS
Diabetes mellitus	35 (39%)	6 (27%)	NS
Chronic renal failure	5 (6%)	2 (9%)	NS
Cirrhosis	1 (1%)	2 (9%)	NS
Previous cancer:	2 (2%)	2 9%)	NS
Hematological malignancy:	1 (1%)	0 (0%)	NS
Immune deficiency	11 (12%)	2 (9%)	NS
*Treatment (N, %)*			
Corticosteroids	90 (100%)	2 (9%)	NS
Tocilizumab	9 (10%)	0 (0%)	NS
*Organ support therapy (N, %)*			
Mechanical ventilation	50 (56%)	8 (28%)	NS
Hemodialysis	9 (10%)	2 (9%)	NS
*Biological data (mean) [min–max]*			
Neutrophil count (G/L)	7.59 [0.78–22.8]	11.55 [2.7–19.4]	0.0047
Lymphocyte count (G/L)	0.76 [0.12–1.87]	0.84 [0.16–1.51]	NS
CRP (mg/L)	152 [0–428]	253 [0–418]	0.0094
Fibrinogen (g/L)	6.9 [4.84–9]	6.61 [1.29–10.2]	NS

Sex, risk factors, and type of treatment were compared with the *χ*^2^ test. The Mann–Whitney test was used to compare quantitative variables

Biological data have been measured at the admission of patients to ICU (Day 1)

*SOFA* Sequential Organ Failure Assessment, *CRP* C-reactive protein, *NS* not significant

### Circulating neutrophils from COVID-19 patients are hyperactivated and bind to platelets

**Fig. 1:**
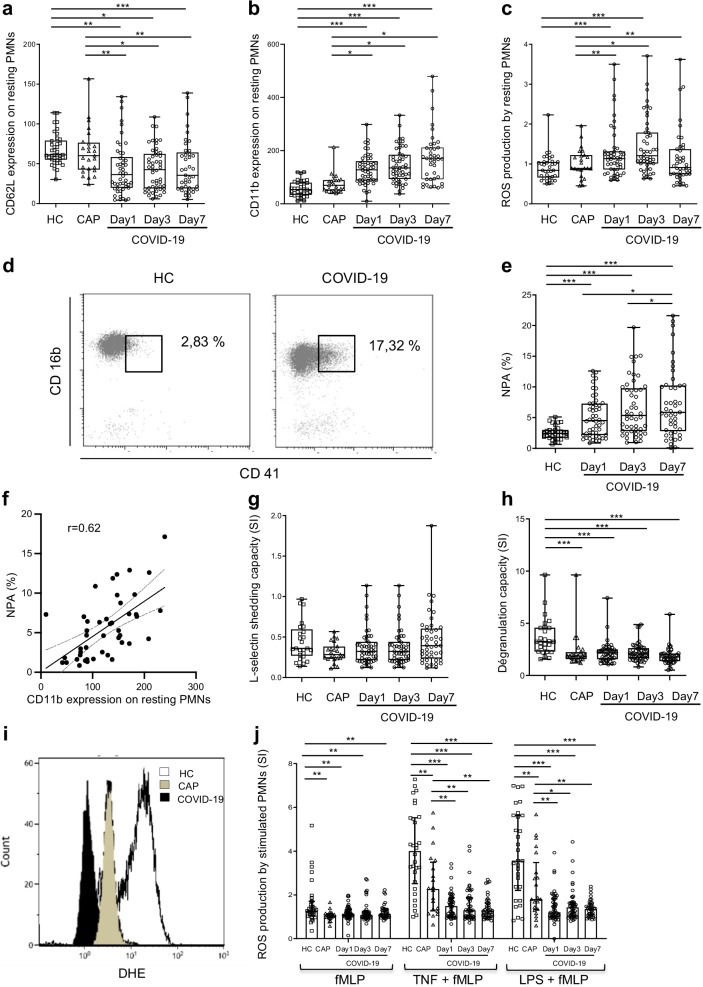
Phenotypic and functional characterization of circulating neutrophils from COVID-19 patients. **a**, **b** Surface expression of CD62L (**a**) and CD11b (**b**) on resting neutrophils (PMNs) was studied in whole-blood samples maintained at 4 °C and stained with specific monoclonal antibodies. Results are expressed as mean fluorescence intensity (MFI). **c** Production of ROS by unstimulated neutrophils was studied with dihydroethidium (DHE) oxidation after treatment of whole-blood samples for 50 min with PBS; results are expressed as MFI. **d**–**f** Analysis of neutrophil-platelet aggregates (NPA). **d** Gating strategy and representative dot plots of flow cytometry analysis. **e** NPA levels, expressed as percentage of neutrophils that bind platelets. **f** Correlation of the percentage of NPA with CD11b expression at the neutrophil surface. **g**, **h** Analysis of capacity for L-selectin shedding (**g**) and neutrophil degranulation (**h**) in response to stimulation. CD62L and CD11b expression were analyzed after incubation of whole-blood samples for 45 min with PBS or fMLP (10^−6^ M). Results are expressed as a stimulation index (SI; MFI of stimulated sample/MFI of unstimulated sample). **i**, **j** ROS production by stimulated neutrophils was measured after pretreatment of whole-blood samples for 45 min with PBS, TNF-α (TNF, 5 ng/mL) or LPS (TLR4 agonist, 10 ng/mL). One histogram representative of ROS production by LPS-primed samples from a control (white), a CAP patient (grey) and a COVID-19 patient (black) (**i**). Results are expressed as SI (**j**). Samples came from age-matched healthy controls (HCs) (*n* = 38), CAP patients (*n* = 22) and COVID-19 patients at day 1 (*n* = 53), day 3 (*n* = 49) and day 7 (*n* = 40). Values are means ± SEM. *P < 0.05, **P < 0.01, ***P < 0.001, adjusted for age

From day 1 to day 7, expression of CD62L decreased and that of CD11b increased on resting neutrophils from patients with COVID-19, in comparison with neutrophils from HCs and CAP patients (Fig. 1a and b). This finding indicates the basal hyperactivation of the COVID-19 patients' circulating neutrophils. The serum level of granule-derived proteins released by activated neutrophils, e.g., NE and LTB4 (Additional file 1: Fig. S1a and b) also rose in these patients, compared with HCs, and thus confirmed their phenotype of enhanced circulating neutrophil degranulation. Because neutrophils are reported to trigger microbicidal mechanisms on activation [24], we measured neutrophil production of ROS and found its basal production by unstimulated neutrophils slightly higher in the COVID-19 patient group than in HCs and CAP patients (Fig. 1c).

Activated neutrophils from COVID-19 patients can interact with platelets via CD11b/CD18 [25, 26]. Consistently with these reports, we found higher circulating levels of neutrophil-platelet aggregates (NPAs) in the COVID-19 patient group than in HCs (Fig. 1d and 1e), positively correlated with CD11b expression at the surface of neutrophils from COVID-19 patients (Fig. 1f).

### Reduced functional responses of circulating neutrophils from COVID-19 patients

Optimal stimulation with the bacterial peptide fMLP induced normal L-selectin shedding in the COVID-19 patient group (Fig. 1g). In contrast, fMLP-induced CD11b translocation was significantly lower in COVID-19 patients and CAP patients than in HCs (Fig. 1h). We then assessed ROS production capacity of neutrophils in response to various stimuli. We have previously reported that neutrophils in whole blood produce minimal ROS in response to a single stimulus [27, 28]. We therefore studied neutrophil oxidative burst in response to the bacterial peptide formyl-methionyl-leucyl-phenyl-alanine (fMLP) after priming with TNF-α or TLR4 agonists. ROS production by non-primed neutrophils was significantly lower in CAP and COVID-19 patients than in HCs. Under TNF and LPS priming conditions followed by fMLP stimulation, ROS production from day 1 to day 7 was much lower in both COVID-19 and CAP patients than in HCs and, importantly, significantly lower in COVID-19 than CAP patients (Fig. 1i and j). We also analyzed the capacity of neutrophils from COVID-19 patients to produce ROS after priming with TLR8 agonist to directly address the importance of neutrophil sensing of SARS-CoV-2 RNA [29] in the regulation of ROS production. Oxidative burst was strongly impaired in COVID-19 patients compared with HCs (Additional file 1: Fig. S1c).

### Impaired neutrophil homeostasis in COVID-19 patients

In accordance with previous studies performed during the first COVID-19 wave [30–32], we observed the presence of a heterogenous population of mature and immature (CD16^low^CD10^low^) neutrophils in COVID-19 patients (Additional file 1: Fig. S1d). No correlation was found between the decrease in ROS production in priming conditions and the percentage of immature neutrophils (*P* = 0.32 and *P* = 0.077 for TNF and LPS priming respectively).

**Fig. 2:**
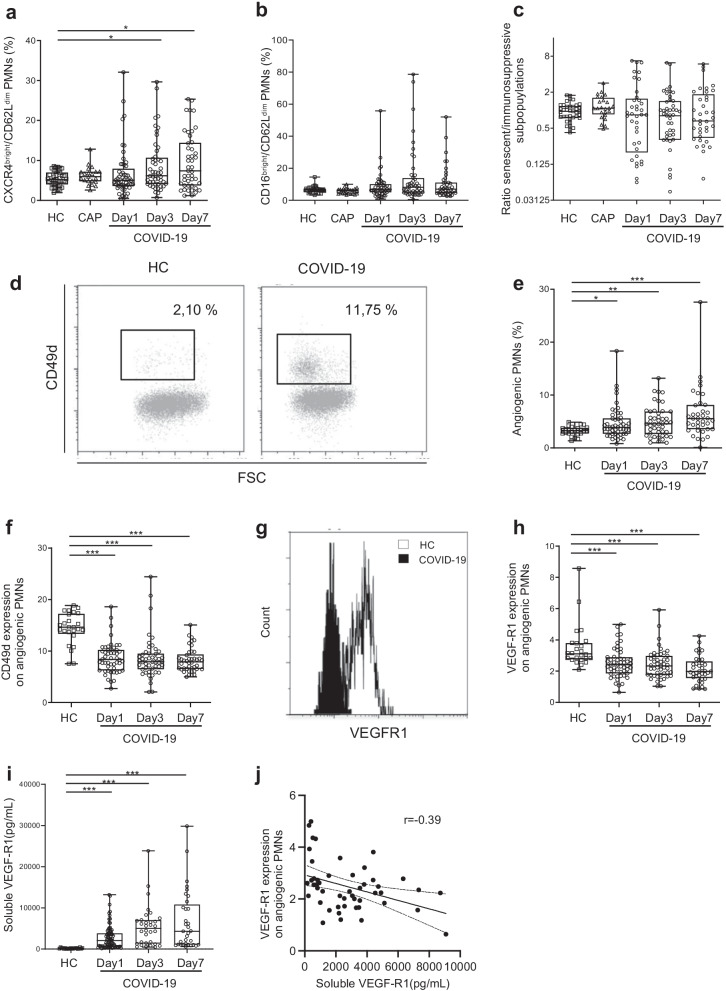
Impaired homeostasis of circulating neutrophils in COVID-19 patients. **a**–**c** Analysis of senescent and immunosuppressive neutrophil subsets in COVID-19 patients. Whole-blood samples were incubated for 45 min at 4 °C with Pe-Cy7-anti-human CXCR4, PE-anti-human CD11b, and APC-anti-human CD62L (**a**) or with FITC-anti-human CD16, PE-anti-human CD11c, Pe-Cy7-anti-human CD11b, and APC-anti-human CD62L (**b**) antibodies. **a** Percentages of the CXCR4^bright^/CD62L^dim^ senescent PMN subset. **b** Percentages of the CD16^bright^/CD62L^dim^ immunosuppressive PMN subset. (**c**) Ratio between the senescent and the immunosuppressive PMN subsets. **d**–**h** Analysis of the angiogenic neutrophil subset in COVID-19 patients. Whole-blood samples were incubated for 45 min at 4 °C with FITC-anti-human VEGF-R1 and BV-481-anti-human CD49d. **d** Representative dot plot of angiogenic CD49d^bright^ neutrophils gated according to forward-scattered light (FSC)/CD49d expression. **e** Percentages of the CD49d^bright^ angiogenic neutrophil subset. **f** Expression of CD49d expression was analyzed at the surface of angiogenic neutrophils (CD49d^bright^); results are expressed in MFI. **g** One histogram representative of VEGF-R1 expression on angiogenic neutrophils from a control (white) and a COVID-19 patient (black). **h** Expression of VEGF-R1 on angiogenic neutrophils; results are expressed as MFI. Samples came from age-matched healthy controls (HCs) (*n* = 38), CAP patients (*n* = 22) and COVID-19 patients at day 1 (*n* = 53), day 3 (*n* = 49) and day 7 (*n* = 40). **i** Soluble VEGF-R1 was quantified by ELISA in HCs and COVID-19 patients at day 1 (*n* = 82), day 3 (*n* = 33) and day 7 (*n* = 32); results are as pg/ml. Values are means ± SEM. **j** Correlation between expression of VEGF-R1 on angiogenic neutrophils and soluble VEGF-R1 in COVID-19 patients. *P < 0.05, **P < 0.01, ***P < 0.001, adjusted for age. Samples came from age-matched healthy controls (HCs) (*n* = 38), CAP patients (*n* = 22) and COVID-19 patients at day 1 (*n* = 53), day 3 (*n* = 49) and day 7 (*n* = 40)

The percentage of the senescent CXCR4^high^/CD62L^low^ neutrophil subset was slightly higher at day 3 and day 7 in COVID-19 patients than in HCs (Fig. 2a), whereas the percentage of the CD16^dim^/CD62L^bright^ immunosuppressive subset, reported to exhibit reduced proinflammatory properties [33, 34], as well as the ratio of senescent to immunosuppressive subsets did not significantly differ between patients and HCs from inclusion to day 7 (Fig. 2b and c). The percentage of senescent neutrophil subset did not differ between patients who received and patients who did not receive tocilizumab.

Among CXCR4^high^ aged neutrophils, a specific subpopulation of CD49b^high^ and VEGF-R1^high^ neutrophils has pro-angiogenic properties [9] and is reported to migrate to hypoxic tissue and to participate in neovascularization [10]. Unexpectedly, we observed an expansion in the circulation of this angiogenic neutrophil subset in COVID-19 patients at day 1 (Fig. 2d and e) and persisting at day 3 and day 7 (Fig. 2e), together with a decrease in the expression of CD49b (Fig. 2f) and VEGF-R1 (Fig. 2g and h) on the surface of angiogenic neutrophils. Because proteolytic cleavage of VEGF-R1 from lung epithelial cell surface has been reported during ARDS [35], we measured the level of soluble VEGF-1-receptor1 (sVEGF-R1) and found higher sVEGF-R1 levels in the COVID-19 patient group from day 1 to day 7 than in HCs (Fig. 2i). Accordingly, sVEGF-R1 levels were negatively correlated with VEGF-R1 expression at the neutrophil surface in COVID-19 patients (Fig. 2j).

### The proportion of circulating reverse-migrated neutrophils is highest in COVID-19 patients

**Fig. 3:**
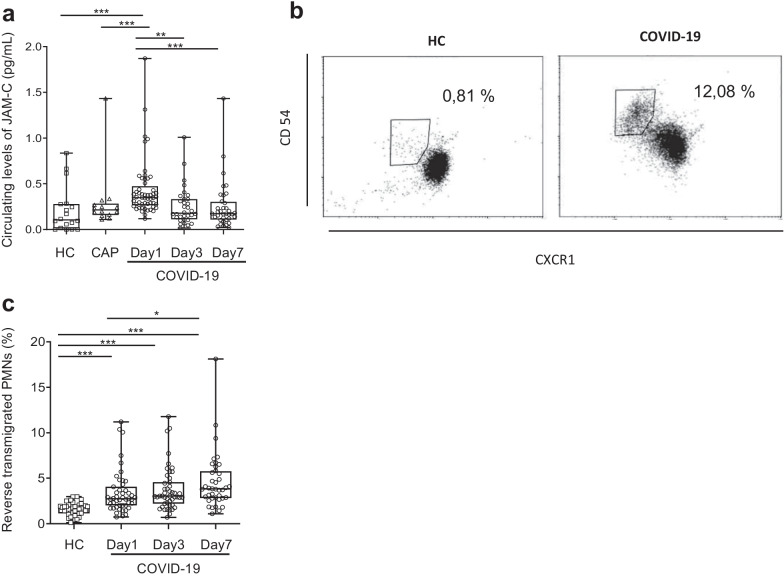
COVID-19 patients have higher levels of soluble JAM-C and of neutrophil reverse transendothelial transmigration. **a** Soluble JAM-C (sJAM-C) was quantified by ELISA; results are expressed as pg/ml. **b**, **c** Quantification of neutrophils undergoing reverse-endothelial transmigration (reverse transmigrated neutrophils, rTEM). Whole-blood samples were incubated for 45 min at 4 °C with FITC-anti-human CD181 (CXCR1) and PE-anti-human CD54 antibodies. **b** Representative dot plots of the neutrophil phenotype according to CXCR1 and CD54 expression in an HC (left) and a COVID-19 patient (right); **c** Percentage of rTEM neutrophils. Samples came from age-matched healthy controls (HCs) (*n* = 38), CAP patients (*n* = 22), and COVID-19 patients at day 1 (*n* = 53), day 3 (*n* = 49), and day 7 (*n* = 40). Values are means ± SEM. *P < 0.05, **P < 0.01, ***P < 0.001, adjusted for age

The LTB4-NE axis is reported to induce cleavage of endothelial JAM-C, which plays a role in tight junction formation, leukocyte adhesion, and transendothelial migration: Proteolytic cleavage of endothelial JAM-C leading to soluble JAM-C (sJAM-C) has been reported to be instrumental in promoting neutrophil rTEM in vivo [36]. Circulating JAM-C levels were significantly higher at day 1 in COVID-19 patients than in HCs and CAP patients (Fig. 3a). Accordingly, we found a higher percentage of rTEM neutrophils in COVID-19 patients at day 1, day 3, and day 7 than in HCs (Fig. 3b and c).

### Neutrophil abnormalities are associated with vascular inflammation in COVID-19 patients

**Fig. 4:**
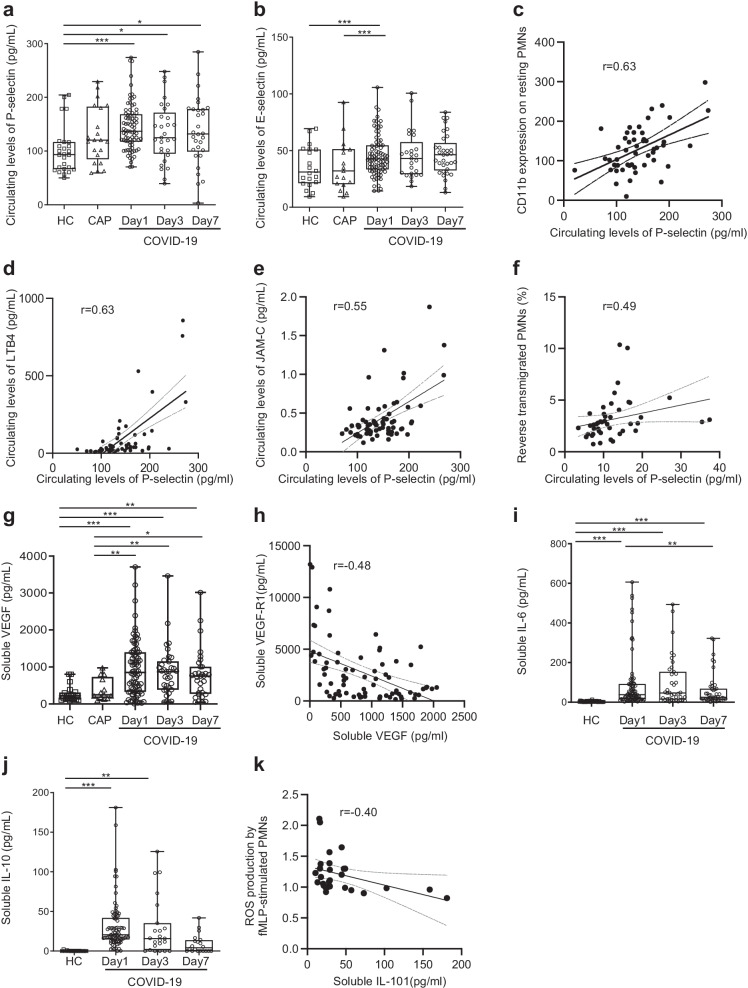
Neutrophil abnormalities are associated with vascular inflammation in COVID-19 patients. **a**–**f** Evaluation of soluble markers of endothelial activation. Levels of soluble P-selectin (**a**) and E-selectin (**b**) were quantified by ELISA; results are expressed as pg/ml. Correlation between soluble P-selectin and CD11b expression on neutrophils (**c**), circulating LTB4 levels (**d**), circulating JAM-C level (**e**) and the percentage of rTEM neutrophils (**f**). **g**–**k** Measurement of circulating levels of cytokines. Soluble VEGF was quantified by ELISA; results are as pg/mL (**g**). Correlation between soluble VEGF and VEGF-R1 (**h**). IL-6 (**i**), and IL-10 (**j**) were quantified by ELISA; results are as pg/ml. Correlation between soluble IL-10 and ROS production by fMLP-stimulated neutrophils (**k**). All samples came from age-matched healthy controls (HC, *n* = 40) and COVID-19 patients at day 1 (*n* = 53), day 3 (*n* = 49) and day 7 (*n* = 40). Values are means ± SEM. *Significantly different from controls *P* < 0.05, **P < 0.01, ***P < 0.001, adjusted for age

Each membrane-bound endothelial selectins has a soluble form that can be measured in the plasma and is used as a marker of endothelial injury and vascular inflammation [37, 38]. At inclusion, COVID-19 patients had higher soluble P- and E-selectin levels than the HCs (Fig. 4a and b). Furthermore, soluble P-selectin levels in COVID-19 patients were positively correlated with markers of neutrophil activation, i.e., CD11b expression (Fig. 4c) and LTB4 (Fig. 4d), as well as the circulating levels of JAM-C (Fig. 4e) and the percentage of rTEM neutrophils (Fig. 4f). We next measured soluble levels of VEGF, which is reported to be critical in the regulation of both vascular permeability and endothelial cell survival [39], and observed higher circulating levels of sVEGF in COVID-19 patients as compared to than in HCs and CAP patients (Fig. 4g). Consistent with the fact that soluble VEGF-R1 is a physiological antagonist of VEGF [40], sVEGF levels were negatively correlated with sVEGF-R1 in COVID-19 patients (Fig. 4h).

As expected [41], circulating levels of IL-6 and IL-10 were higher in ICU COVID-19 patients (Fig. 4i and j). IL-6 levels were significantly lower at day 7 as compared to those at day 1. Regarding IL-10, we observed a trend to decline during patient follow-up, although no significant difference was noted between the COVID-19 groups. At day 1, the ROS production in response to fMLP correlated negatively with that of IL-10 (Fig. 4k). Circulating TGFβ levels did not significantly differ between patients and healthy controls from inclusion to day 7 (not shown).

### Neutrophil abnormalities are associated with clinical severity of COVID-19

To investigate the relation between the neutrophil markers and lung or other organ failures in COVID-19, we distinguished respiratory from non-respiratory SOFA scores. Unlike some previous studies, neutrophil counts in COVID-19 patients at ICU admission did not correlate with the SOFA, the respiratory SOFA and the non-respiratory SOFA scores (Additional file 1: Fig. S2a, b, c). However, neutrophil counts in COVID-19 patients at day 3 and day 7 post-admission correlate with the SOFA scores measured at the same time (Additional file 1: Fig. S2d–i).

At ICU admission, the neutrophil surface CD62L expression was not significantly associated with the non-respiratory SOFA score (Additional file 1: Fig. S3a) but was negatively associated with high global SOFA and respiratory SOFA scores after adjustment for demographic and laboratory variables (Additional file 1: Fig. S3b and c). In addition, higher circulating of LTB4 and NE, two neutrophil hyperactivation markers, was positively associated with high global SOFA and respiratory SOFA scores (Additional file 1: Fig. S3d and e). Higher percentage of senescent and immunosuppressive subsets were positively associated with respiratory SOFA score (Additional file 1: Fig. S4f and g). We also observed a negative association between VEGF-R on angiogenic neutrophil surfaces and a high global SOFA score at ICU admission of COVID-19 patients (Additional file 1: Fig S3h). Finally, the percentages of rTEM and angiogenic neutrophil subsets analyzed at day 3 were positively associated with the respiratory SOFA score calculated at the same time (Additional file 1: Fig S3i and j).

In accordance with previous data [42], we found a positive association between IL-10 measured at inclusion and global SOFA at day 1 (Additional file 1: Fig. S4b) as well as between IL-6 and IL-10 measured at inclusion and global SOFA at day 7 (Additional file 1: Fig. S4c and d).

### COVID-19 patients who died had higher percentage of angiogenic neutrophil subset and greater impairment of neutrophil oxidative burst than survivors did

**Fig. 5:**
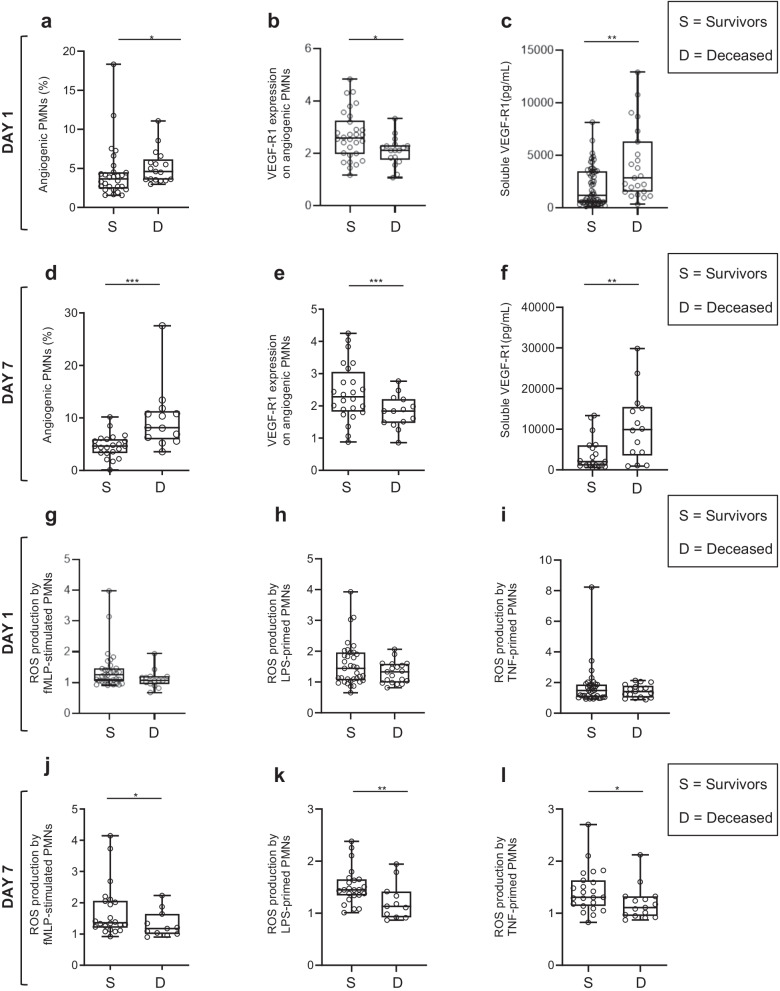
COVID-19 patients who died had higher percentage of angiogenic neutrophils and greater impairments of neutrophil oxidative burst than survivors did. **a**–**f** Analysis of the angiogenic neutrophil subset in COVID-19 patients. Percentages of the CD49d^bright^ angiogenic neutrophil subset measured at day 1 (**a**) and day 7 (**d**). Expression of VEGF-R1 on angiogenic neutrophils measured at day 1 (**b**) and day 7 (**e**); results are expressed as MFI. Soluble VEGF-R1 was quantified by ELISA at day 1 (**c**) and day 7 (**f**); results are expressed as pg/mL. **g**–**l** ROS production in response to fMLP by unprimed-neutrophils at day 1 (**g**) and day 7 (**j**), by LPS-primed neutrophils at day 1 (**h**) and day 7 (**k**), and TNFα-primed neutrophils at day 1 (**i**) and day 7(**l**). All measurements came from deceased COVID-19 patients or survivors at day 60 post-ICU inclusion. Values are means ± SEM. Statistical significance as determined by the nonparametric Mann–Whitney test is indicated. *Significantly different *P* < 0.05, **P < 0.01, ***P < *0.001,* adjusted for age

COVID-19 patients were classified into two groups according to their outcome at day 60. The COVID-19 patients who died had significantly higher percentage of circulating angiogenic neutrophils (Fig. 5a) as well as lower expression of VEGF-R1 (Fig. 5b) associated with higher soluble VEGF-1 (Fig. 5c) at day 1, than survivors. Similar results were observed at day 7 (Fig. 5d-f). At COVID-19 patients' admissions to the ICU, ROS production in response to fMLP by unprimed, LPS-primed or TNFα-primed neutrophils did not differ between these two groups (Fig. 5g-i) but was lower at day 7 in patients who died than survivors (Fig. 5j-l). In parallel, the COVID-19 patients who died had significantly higher levels of soluble IL-10, an anti-inflammatory cytokine reported to inhibit ROS production by activated neutrophils [43] (Additional file 1: Fig. S5a). In contrast, the patients who died and those who survived did not differ for neutrophil count, neutrophil basal activation state, percentages of immature, senescent and immunosuppressive subsets, or soluble levels of various proinflammatory mediators (Additional file 1: Fig. S5b–p).

As 74% of the deceased patients who died had superinfections during their hospitalization, we analyzed the neutrophil markers according to the occurrence of bacterial or fungal superinfections. ROS production by fMLP-stimulated neutrophils, measured at day 1 and day 7, was significantly lower, in superinfected than non-superinfected COVID-19 patients (Additional file 1: Fig. S6a and d). Moreover, at day 7, TNF and LPS-primed neutrophils produced significantly fewer ROS in superinfected than non-superinfected COVID-19 patients (Additional file 1: Fig. S6e and f).

## Discussion

This extensive investigation of the phenotype and function of peripheral neutrophils from 90 COVID-19 ICU patients used whole blood to minimize any potential bias related to isolation procedures and take the proinflammatory cytokine environment into account. Our results demonstrated that these patients' circulating neutrophils showed continuous basal hyperactivation from their admission to the ICU—hyperactivation not evidenced in CAP patients. The positive association of lower L-selectin expression and higher circulating levels of NE and LTB4 with the respiratory SOFA score suggests that neutrophil hyperactivation might be involved, at least in part, in lung dysfunction. Our critically ill COVID-19 patients had a median SOFA score of 3.5, reflecting a predominantly isolated respiratory failure, showing that the SARS-CoV-2 infection itself rather than the multiorgan dysfunction associated with severe forms triggers these neutrophil function modifications. SARS-CoV-2 infects human cells by attaching to angiotensin-converting enzyme 2 (ACE2) expressed on the epithelial cell lining of the lungs, arteries, heart, kidneys, and intestines. Although neutrophils do not express ACE2, a recent report described their expression of CD147 [44], which was recently shown to act as a receptor for SARS-CoV-2 in cell lines of epithelial origin [45]. Moreover, the highly glycosylated nature of the SARS-CoV-2 spike protein [46] increases its likelihood of binding to CD147, which has three Asn glycosylation sites [47] at the neutrophil surface. It is thus possible that the virus can attach to the neutrophil surface, where it can induce various cellular programs that lead to cell hyperactivation and exhaustion.

Neutrophil hyperactivation increases the circulating concentrations of granule-derived proteins released by activated neutrophils, e.g., NE which might be involved, at least in part, in the JAM-C cleavage and increased neutrophil reverse transendothelial migration [8]. While these neutrophils' departure from an inflammation site resolves this local inflammation, they may then spread throughout the body via the bloodstream, transmigrating into other organs and contributing to more organ injuries and systemic inflammation [48]. Reverse migrated neutrophils show prolonged lifespans and delayed apoptosis [49], which could contribute to persistent and amplified inflammation.

The positive correlation of higher CD11b expression at the neutrophil surface with higher levels of NPAs and of circulating P-selectin suggests that platelets are in a preactivated state and may thus contribute to microthrombotic complications in severely ill patients. P-selectin is also stored in and expressed by endothelial cells, and its elevated plasma levels in patients might also reflect endothelial cell activation and damage [50]. Consistently with this finding, we found elevated levels of sE-selectin in COVID-19 but not CAP patients. The presence of all these biomarkers speaks in favor of endotheliopathy in our patients, in line with previous studies [51, 52].

Hyperactivation of circulating neutrophils may be involved in the impaired neutrophil oxidative burst observed in COVID-19 patients in response to bacterial formyl peptides and could indicate functional neutrophil tolerance/exhaustion. As ROS production in response to fMLP correlated negatively with that of IL-10 level, this impairment could be related to the effect of IL-10 in downregulating neutrophil activity [43]. Such an impairment might also be related at least in part to the corticosteroid treatment administered to all patients in our COVID-19 cohort [53]. In contrast, very few patients from the CAP cohort received corticosteroids. As neutrophils play a key role in the defense against bacterial and fungal infections, these modifications could contribute to the increased susceptibility to the hospital-acquired bacterial and fungal infections that are emerging as a common secondary complication among COVID-19 patients. In accordance with previous data [54], secondary infections, which are observed in 41% of COVID-19 patients in our cohort, are significantly associated with lower 60-day survival. Moreover, ROS production by TNF and LPS-primed neutrophils in response to formyl peptides was significantly lower in COVID-19 patients who died compared to survivors as well as in superinfected patients compared with non-superinfected patients. Nevertheless, the direct link between the occurrence of superinfections in COVID-19 and lower neutrophil oxidative burst remains to be demonstrated.

Neutrophils are typically regarded as terminally differentiated cells that progress from immature neutrophils in the bone marrow to circulating mature inactive neutrophils that can, upon priming and subsequent activation in inflammatory conditions, extravasate into tissues and fulfill their effector functions. At the end of the spectrum, mature neutrophils in the circulatory system, nearing the end of their lifetime, may acquire a specific phenotype. During follow-up, we observed an increase in the percentage of longer-lived CXCR4^high^ neutrophils in COVID-19 patients. It has been proposed that gut microbiota regulate neutrophil aging [7]. Recent studies report gut dysbiosis in COVID-19 patients [55, 56]. Furthermore, previous intestinal dysbiosis observed in type 2 diabetes, obesity, hypertension, coronary heart disease, and in other age-related disorders are involved in the deregulation of the inflammatory immune response to SARS-CoV-2, which promotes infection, dissemination, and severity in patients with comorbidities [57]. In addition, glucocorticoid signaling in humans is proposed to drive diurnal aging in neutrophils [58].

Neutrophil aging may favor a proinflammatory phenotype, and the presence of aged neutrophils in the circulation may predispose individuals to vascular inflammation, independently of NET formation [59]. The increased percentage of pro-angiogenic neutrophils in blood from COVID-19 patients may be related at least in part to decreased expression of VEGF-R1 at neutrophil surface, a probable consequence of metalloprotease-mediated ectodomain cleavage [60]. Accordingly, we found higher soluble levels of VEGF-R1 in COVID-19 patients, correlated with the decreased expression of VEGF-R1 on the surface of angiogenic neutrophils. This abnormality might limit neutrophil recruitment to hypoxic areas [9] increasing the percentage of circulating angiogenic neutrophils. This subset is characterized by the ability to release high quantities of MMP-9 [9] thought to be involved in the pathogenesis of inflammatory vascular diseases [61]. Importantly, COVID-19 patients who died had significantly higher percentage of circulating angiogenic neutrophils and lower expression of VEGF-R1 on angiogenic neutrophils than survivors. Accordingly, soluble VEGF-R1 was significantly increased in COVID-19 patients who died.

Our study has several limitations. First, the CAP population consists of patients with severe pneumonia driven by multiple pathogens, both bacterial and viral and is not perfectly matched to our COVID-19 patient cohort. The comparison of COVID-19 patients with a pure viral pneumonia cohort could help further specify the unique immune signatures of SARS- CoV-2. Second, we did not perform functional testing of neutrophil subsets. Third, ROS production by neutrophil in response to TLR8 agonist was not investigated in CAP patients. However, our results are based on a large population and include a kinetic analysis that adds to the current knowledge regarding the COVID-19-related impact on neutrophil functions. Future experiments in an independent cohort are required for further validation.

It’s important to note that in our study, all included COVID-19 patients received corticosteroid treatment. While the immunosuppressive effect of steroids is undisputed and desirable in the context of severe COVID-19 [62], corticosteroids might increase the susceptibility to secondary bacterial or fungal infections in vulnerable patients, such as elderly, frail patients and might potentially affect the risk–benefit balance [63–65]. The impairment of neutrophil oxidative burst in response to bacterial formyl peptide highlighted in our COVID-19 cohort emphasizes the resulting immunosuppression due to corticosteroid treatment. Thereby, our results question the systematic use of high dose corticosteroid treatment in particular in vulnerable patient groups and a reduction either of steroid dose or treatment duration may be discussed to limit secondary infection risk.

Furthermore, our data should open new perspectives in the development of innovative immunotherapy strategies. Recently, Crainiciuc et al. identify the Src kinase Fgr as a driver of the pathogenic senescent state of neutrophils, and interference with Fgr protected mice from inflammatory injury in a model of ischemia–reperfusion [66]. In addition, targeting junctional adhesion molecule-C has been reported to improve sepsis-induced acute lung injury by decreasing CXCR4^+^ aged neutrophils [67]. Taking into account these data, our new findings obtained from circulation neutrophils from COVID-19 patients suggest that targeting senescent “aged” neutrophils within inflamed vessels might be a promising therapeutic choice for reducing clinical severity in severe SARS-CoV-2 infection.

## Conclusions

In summary, our study highlights neutrophil hyperactivation and impaired homeostasis during severe COVID-19—both abnormalities that might play a central role in endothelial dysfunction, angiogenesis, and vascular inflammation. This study also demonstrates that neutrophil exhaustion appears to play a central role in the pathogenesis of severe COVID-19 and identifies angiogenic neutrophils as a potential harmful subset involved in fatal outcome.

## Supplementary Information

**Additional file 1.Table S1.** Characteristics of Survivors and non-survivors COVID-19 patients participating in the study. **Fig. S1.** Phenotype and homeostasis of neutrophils in COVID-19. **Fig. S2.** Association between neutrophil count from COVID-19 patients and disease severity. **Fig. S3.** Association between neutrophil parameters and disease severity in COVID-19 patients. **Fig. S4.** Association between neutrophil alterations and soluble cytokine levels from COVID-19 patients with disease severity. **Fig. S5.** Comparison of neutrophil phenotype and homeostasis in COVID-19 patients who died and survivors. **Fig. S6.** Greater impairments of neutrophil oxidative burst in superinfected than non-superinfected COVID-19 patients.

## Data Availability

The datasets used and/or analyzed during the current study are available from the corresponding author on reasonable request.

## Abbreviations

ACE2: Angiotensin-converting enzyme 2
ARDS: Acute respiratory distress syndrome
CAP: Community- acquired pneumonia
COVID-19: Coronavirus disease 2019
CRP: C-reactive protein
fMLP: *N*-Formylmethionyl-leucyl-phenylalanine
HC: Healthy control
HE: Hydroethidin
ICU: Intensive care unit
JAM-C: Junctional adhesion molecule-C
LTB4: Leukotriene B4
MMP-9: Matrix metalloproteinase-9
NE: Neutrophil elastase
NETs: Neutrophil extracellular traps
NPA: Neutrophil–platelet aggregate
PMN: Polymorphonuclear neutrophils
ROS: Reactive oxygen species (ROS)
rTEM: Reverse transendothelial migration
SOFA: Sequential Organ Failure Assessment
TLR: Toll-like receptor
