# Supplementary material for: Impairment of neutrophil functions and homeostasis in COVID-19 patients: association with disease severity
Source: Crit Care. 2022 May 30;26:155. doi: 10.1186/s13054-022-04002-3 (PMC9149678; doi:10.1186/s13054-022-04002-3)
Supplement: Supplementary file 1 — Additional file 1.Table S1. Characteristics of Survivors and non-survivors COVID-19 patients participating in the study. Fig. S1. Phenotype and homeostasis of neutrophils in COVID-19. Fig. S2. Association between neutrophil count from COVID-19 patients and disease severity. Fig. S3. Association between neutrophil parameters and disease severity in COVID-19 patients. Fig. S4. Association between neutrophil alterations and soluble cytokine levels from COVID-19 patients with disease severity. Fig. S5. Comparison of neutrophil phenotype and homeostasis in COVID-19 patients who died and survivors. Fig. S6. Greater impairments of neutrophil oxidative burst in superinfected than non-superinfected COVID-19 patients. [file 13054_2022_4002_MOESM1_ESM.docx]

**Impairment of neutrophil functions and homeostasis in COVID-19 patients:**

**Association with disease severity**

Chloé Loyer^1,2^, Arnaud Lapostolle^1,2^, Tomas Urbina^2,3^, Alexandre Elabbadi^4^, Jean-Rémi Lavillegrand^2,5,6^, Thomas Chaigneau^1,2^, Coraly Simoes^1,2^, Julien Dessajan^2,4^, Cyrielle Desnos^2,4^, Mélanie Morin-Brureau^1,2^, Yannick Chantran^1,2,7^, Pierre Aucouturier^1,2,7^, Bertrand Guidet^2,3^, Guillaume Voiriot^2,4^, Hafid Ait-Oufella^2,3,6^, & Carole Elbim^1,2*^

**Supplementary Materials**

**Determination of neutrophil subsets**

The neutrophil subsets were analyzed with the Gallios flow cytometer. To investigate the senescent CXCR4^bright^/CD62L^dim^ neutrophil subset, whole-blood samples collected on lithium heparinate and kept on ice were incubated for 45 min with PE-Cy7-anti-human CXCR4 (clone 12G5, Sony Biotech, San Jose, CA), PE-anti-human CD11b (clone ICRF44, BD Biosciences), and APC-anti-human CD62L (BD Biosciences) antibodies; for the immunosuppressive CD16^bright^/CD62L^dim^ neutrophil subset, for 45 min with FITC-anti-human CD16 (clone 1D3, Beckman Coulter, Brea, CA), PE-anti-human CD11c (clone 3.9, Sony Biotech), PE-Cy7-anti-human CD11b (clone Bear 1, Beckman Coulter), and APC-anti-human CD62L (BD Biosciences) antibodies; for the angiogenic subset with BV-421-anti-CD49d and FITC-anti-VEGF-R1 (BD Biosciences) antibodies, and for the rTEM neutrophil subset, for 45 min with FITC-anti-human CD81 (BD Biosciences) and PE-anti-CD54 (BD Biosciences) antibodies. To discriminate between immature (CD16^low/high^CD10^–^) and mature (CD16^high^CD10^+^) subsets, samples kept on ice were incubated for 45 min with PE-anti-human CD16b (clone CLB-gran11.5, BD Biosciences) and FITC-anti-human CD10 (clone HI10a, BD Biosciences) antibodies.

The blood was then lysed with BD FACS lysing solution, and the cells were then resuspended with Cell Fix 1X (BD Biosciences) (Dong et al. Ann Neurol. 2018 Feb;83(2):387-405).

**Flow cytometry analysis**

After staining, the blood was then lysed with BD FACS lysing solution, and the cells were then resuspended with Cell Fix 1X (BD Biosciences). Cells were analyzed with a Gallios^TM^ flow cytometer and the data with Kaluza software (Beckman Coulter). Neutrophil expression of surface molecules and ROS production were determined by using forward and side scatter to identify the granulocyte population and to gate out other cells and debris. The purity of the gated cells was assessed by using FITC- or PE-conjugated CD3, CD45, CD14, and CD15 antibodies. Ten thousand events were analyzed per sample, and fluorescence pulses amplified by 4-decade logarithmic amplifiers. In all cases, unstained cells were run, and the photomultiplier settings adjusted so that the unstained cell population appeared in the lower left-hand corner of the fluorescence display. In the multicolor analysis, single-cell controls were used to optimize signal compensation. All results were obtained with the use of a constant photomultiplier gain value.

**Table S1. Characteristics of Survivors and non-survivors COVID-19 patients participating in the study**

| Characteristics | Survivors  N= 70 | Non-survivors  N=20 | P value |
| --- | --- | --- | --- |
| Men (N, %) | 41 (59%) |  | NS |
| Age (years, SD) | 64 ± 11 | 70 ± 8 | <0.0001 |
| SOFA score Day 1 | 3.05 [0-12] | 4.65 [0-11] | 0.0065 |
| Body mass index  <30  >30 | 34 (49%)  35 (50%) | 13 (65%)  5 (25%) | NS  NS |
| *Comorbidity (N, %)* |  |  |  |
| Arterial hypertension | 46 (66%) | 14 (70%) | NS |
| Diabetes mellitus | 25 (36%) | 10 (50%) | NS |
| Chronic renal failure | 1 (1%) | 4 (20%) | 0.0038 |
| Cirrhosis | 1 (1%) | 0 (0%) | NS |
| Previous cancer: | 1 (1%) | 1 (5%) | NS |
| Hematological malignancy: | 0 (0%) | 1 (5%) | NS |
| Immune Deficiency | 9 (10%) | 2 (2%) | NS |
| *Treatment (N, %)* |  |  |  |
| Corticosteroids | 70 (100%) | 20 (100%) | NS |
| Tocilizumab | 9 (13%) | 0 (0%) | NS |
| *Organ support therapy (N, %)* |  |  |  |
| Sedative drugs | 31 (44%) | 17 (85%) | 0.0018 |
| Mechanical ventilation | 32 (46%) | 18 (90%) | <0.0001 |
| Prone positioning | 30 (43%) | 17 (85%) | 0.0009 |
| Hemodialysis | 4 (6%) | 5 (25%) | 0.0234 |
| *Biological data (mean) [min-max]* |  |  |  |
| Neutrophil count (G/L) | 7.63 [1.88-22.8] | 7.52 [0.78-15.4] | NS |
| Lymphocyte count (G/L) | 0.78 [0.3-1.87] | 0.70 [0.12-1.57] | NS |
| CRP (mg/L) | 157 [0-428] | 146 [23-402] | NS |
| Fibrinogen (g/L) | 6,4 [5,65-9] | 6,95 [4,84-7,9] | NS |
|  |  |  |  |

Abbreviations: SOFA, Sequential Organ Failure Assessment; CRP, C-reactive protein; NS, not significant.

Sex, risk factors, and type of treatment were compared with the χ2 test. The Mann-Whitney test was used to compare quantitative variables.

**Fig. S1. Phenotype and homeostasis of neutrophils in COVID-19**

**a, b** Circulating levels of NE (**a**) and LTB4 (**b**) were quantified by ELISA in healthy controls (HCs) and COVID-19 patients. Results are expressed as pg/mL.

**c** ROS production after TLR8 priming in HCs and COVID-19 patients. ROS production by stimulated neutrophils was measured after pretreatment of whole-blood samples for 45 minutes with TLR8 agonist (ssRNA with 6 UUGU repeats/LyoVec™, 10 ng/mL). Results are expressed as a stimulation index (SI; MFI of stimulated sample/MFI of unstimulated sample)

**d** Percentage of circulating immature neutrophiles in HCs and COVID-19 patients. Whole-blood samples were incubated for 45 minutes at 4°C with anti-human CD16b and anti-human CD10 antibodies.

All samples came from age-matched HCs (n=38) and COVID-19 patients at day 1 (n=53), day 3 (n=49) and day 7 (n=40). Values are means ± SEM. ^*^Significantly different from controls *P*<0.05, ***P*<0.01, ****P*<0.001, adjusted for age.

**Fig. S2. Association between neutrophil count from COVID-19 patients and disease severity**

**a, b, c** Correlation between neutrophil count from COVID-19 patients at ICU admission with their SOFA score (**a**), respiratory SOFA score (**b**) and non-respiratory SOFA score (**c**) at the same time

**d, e, f** Correlation between neutrophil count from COVID-19 patients at day 3 post-admission with their SOFA score (**d**), respiratory SOFA score (e) and non-respiratory SOFA score (**f**) at the same time

**g, h, i** Correlation between neutrophil count from COVID-19 patients at day 7 with their SOFA score (**g**), respiratory SOFA score (**h**) and non-respiratory SOFA score (**i**) at the same time

All samples came from COVID-19 patients at at day 1 (n=53), day 3 (n=49) and day 7 (n=40).

**Figure S3. Association between neutrophil parameters and disease severity in COVID-19 patients.**

**a** Correlation between CD62L expression at the neutrophil surface and non-respiratory SOFA score at ICU inclusion

**b, c** Correlation between CD62L expression on the surface of resting neutrophils from COVID-19 patients at ICU admission and global SOFA (**a**) and respiratory SOFA scores (**b**)

**d, e** Correlation between circulating levels of LTB4 (**c**) and neutrophil elastase (**d**) from COVID-19 patients at ICU admission and global SOFA score.

**f, g** Correlation between the percentage of the CXCR4^bright^/CD62L^dim^ senescent PMN subset (**e**) and the percentage of the CD16^bright^/CD62L^dim^ immunosuppressive PMN subset (**f**) from COVID-19 patients at ICU admission and respiratory SOFA score.

**h** Correlation between VEGF-R expression (MFI) at the surface of angiogenic neutrophils from COVID-19 patients at ICU admission and the global SOFA score.

**i** Correlation between the percentage of reverse transmigrated neutrophils in COVID-19 patients 3 days post-inclusion and respiratory SOFA calculated at the same time

**j** Correlation between the percentage of angiogenic neutrophils in COVID-19 patients 3 days post-inclusion and respiratory SOFA calculated at the same time.

Samples came from COVID-19 patients at day 1 (n=53) and day 3 (n=49). Results are adjusted for age.

**Fig. S4. Association between neutrophil alterations and soluble cytokine levels from COVID-19 patients with disease severity.**

**a** Correlation between IL-10 level measured at ICU inclusion and global SOFA score at the same time

**b, c** Correlation between IL-6 (**c**) and IL-10 (**d**) levels measured at ICU inclusion and global SOFA score at day 7

All samples came from age-matched HCs (n=38) and COVID-19 patients at day 1 (n=53) and day 7 (n=40).

**Fig. S5. Comparison of neutrophil phenotype and homeostasis in COVID-19 patients who died and survivors**

**a** Soluble levels of IL-10 were quantified by ELISA. **b** Neutrophil count. **c, d** Surface expression of CD62L and CD11b on resting neutrophils; results are expressed as MFI. **e** ROS production by unstimulated neutrophils; results are expressed in MFI. **f, g, h, i** Percentages of the immature CD10^low^, the CXCR4^bright^/CD62L^dim^ senescent, the CD16^bright^/CD62L^dim^ immunosuppressive PMN, and the rTEM CD54^high^, CXCR1^low^ subsets. **j, k, l, m, n** Soluble VEGF, JAM-C, E-selectin, P-selectin and IL-6 were quantified by ELISA; results are expressed as pg/ml. **o, p** Circulating level of CRP and Fibrinogen.

All measurements were performed at ICU inclusion and came from deceased COVID-19 patients or survivors at day 60 post-ICU inclusion. Values are means ± SEM. Statistical significance as determined by the nonparametric Mann-Whitney test is indicated. ^*^Significantly different *P*<0.05, adjusted for age.

**Fig. S6.** **Greater impairments of neutrophil oxidative burst in superinfected than non-superinfected COVID-19 patients**

ROS production in response to fMLP by unprimed neutrophils at day 1 (**a**) and day 7 (d), by LPS-primed neutrophils at day 1 (**b**) and day 7 (**e**), and TNFα-primed neutrophils at day 1 (**c**) and day 7 (**f**).

All measurements came from superinfected COVID-19 patients (measurements performed at day 1, n=17 and measurements performed at day 7, n=16) or nonsuperinfected during ICU stay (measurements performed at day 1, n=33 and measurements performed at day 7, n=24). Values are means ± SEM. Statistical significance as determined by the nonparametric Mann-Whitney test is indicated. ^*^Significantly different *P*<0.05, ***P*<0.01, adjusted for age.
